# Polyhedral [M_2_B_5_] Metallaborane Clusters and Derivatives: An Overview of Their Structural Features and Chemical Bonding

**DOI:** 10.3390/molecules25143179

**Published:** 2020-07-12

**Authors:** Rini Prakash, Jean-François Halet, Sundargopal Ghosh

**Affiliations:** 1 Department of Chemistry, Indian Institute of Technology Madras, Chennai 600036, India; rini.prakash1992@gmail.com; 2 Univ Rennes, CNRS, Institut des Sciences Chimiques de Rennes, UMR 6226, F-35000 Rennes, France

**Keywords:** dimetallaborane, dimetallapentaborane, electron count, oblate clusters, boron

## Abstract

A large number of metallaborane clusters and their derivatives with various structural arrangements are known. Among them, M_2_B_5_ clusters and derivatives constitute a significant class. Transition metals present in these species span from group 4 to group 7. Their structure can vary from oblatonido, oblatoarachno, to arachno type open structures. Many of these clusters appear to be hypoelectronic and are often considered as ‘rule breakers’ with respect to the classical Wade–Mingos electron counting rules. This is due to their unique highly oblate (flattened) deltahedral structures featuring a cross-cluster M−M interaction. Many theoretical calculations were performed to elucidate their electronic structure and chemical bonding properties. In this review, the synthesis, structure, and electronic aspects of the transition metal M_2_B_5_ clusters known in the literature are discussed. The chosen examples illustrate how, in synergy with experiments, computational results can provide additional valuable information to better understand the electronic properties and electronic requirements which govern their architecture and thermodynamic stability.

“It is the intrinsic electronic structure of boron that fosters cluster generation and it continues to do so when combined with transition metals”.

[1]

## 1. Introduction

Chemists know how the electronic structure and geometry of molecules are strongly imbricated. In essence, the electron count governs the shape of molecules because it relates to the closed-shell principle [2]. This principle states that, in general, any molecule is (thermodynamically and kinetically) stable when a significant energy gap separates the fully occupied molecular orbitals (MO) (generally the bonding or nonbonding ones) from the unoccupied MOs (generally the antibonding ones). Based on this, different electron counting rules have been derived, such as the octet and the 18-electron rules which are the simplest and the more widespread ones that many main group and transition metal compounds conform to. These rules are of pivotal importance, providing a rudimentary means to describe the chemical bonding in terms of localized two-center−two-electron (2c−2e) bonds. However, these electron counting rules are not universal and often have a limited scope of application. For instance, the octet and 18-electron rules become insufficient to describe the bonding in systems that have three-dimensional (3-D) structures where extensive delocalization occurs. Hence, new electron counting rules and models have then emerged to rationalize these compounds by the use of multicenter bonding descriptions, of which the three-center−two-electron (3c−2e) model is the simplest. This is particularly the case of many boron-containing 3-D molecules. Such systems containing boron obey the closed-shell principle, but not as in molecules made of its neighbors, carbon or silicon. Boron is somewhat unique with four atomic orbitals having only three valence electrons. Its “electron deficiency” character governs its rich chemistry, reflecting the numerous ways in which it seeks to solve the problem of having fewer electrons than atomic orbitals available for bonding. This generally results in many boron-to-boron or boron-to-other-element bonding contacts, as exemplified in the electron-deficient boranes.

Nearly from the beginning of borane chemistry, it was realized that localized 2c−2e bonding was not efficient to describe the electronic structure and bonding of boranes and derivatives [3]. With the efforts of K. Wade and others, new electron counting rules were proposed to rationalize the structure of the deltahedral (polyhedra with exclusively triangular faces) borane clusters with respect to their skeletal electron pairs (sep) [4,5]. New terms, such as, closo, nido, or arachno, were then introduced to describe their structures. The structure of the cluster is described by the prefix closo when all its *n* deltahedral vertices are occupied by boron atoms. Such molecules are characterized by *n* + 1 seps. The cluster becomes a nido species, if one vertex of the characteristic deltahedron is vacant and possesses *n* + 2 seps if *n* represents the number of occupied vertices. With two or three vacant vertices in the deltahedron, the clusters are described as arachno or hypho having *n* + 3 or *n* + 4 seps, respectively. Thus, an octahedral cluster such as [B_6_H_6_]^2−^ is a closo species (7 seps), whereas a square-pyramidal cluster, i.e., an octahedron with one unoccupied vertex is a nido species (B_5_H_9_ with 7 seps is an example). Further, a butterfly-like shape encountered in B_4_H_10_ (7 seps) which can be derived from an octahedron with two adjacent vacant vertices corresponds to an *arachno* structure. Alternatively, a square molecule can also be described as an *arachno*-type cluster, i.e., an octahedron with two opposite (i.e., antipodal) vacant vertices.

Later, the powerful concept of the isolobal analogy developed by Hoffmann et al. [6,7] could explain why these electron counting rules, later known as the Wade–Mingos rules (also known as the polyhedral skeletal electron pair theory, PSEPT) [8,9,10], initially established for boranes, could apply successfully to transition metal clusters. Though a transition metal atom and a main group atom are not exactly comparable due the absence valence *d* orbitals in the latter, there were no obvious geometrical consequences due to this. For example, both [B_6_H_6_]^2−^ and [Ru_6_(CO)_18_]^2−^ [11] adopt a closo octahedral structure with seven seps. Note that, organometallic chemists often use the cluster valence electron (cve) count in addition to the sep count. The cve count was obtained by adding the valence electrons of the cluster atoms and the electrons brought by the surrounding ligands [1,12]. Thus, the 7-sep closo-octahedral species [B_6_H_6_]^2−^ and [Ru_6_(CO)_18_]^2−^ possess 26 [6 × 3 (B) + 6 × 1 (H) + 2 (charge)] and 86 [6 × 8 (Ru) + 18 = × 2 (CO) + 2 (charge)] cves, respectively. This suggests that mixed main-group–transition metal clusters can also be rationalized with the isolobal analogy and the same electron counting rules. For instance, both the *closo*-[B_10_H_10_]^2−^ borane [13,14,15] (11 seps or 42 cves) and the metallaborane cluster (CpNi)_2_B_8_H_8_ (Cp = C_5_H_5_) [16] (11 seps or 62 cves) adopt the same ten-vertex bicapped square-antiprismatic closo structure. Hence, the formal replacement of two BH units—one four-connect and one five-connect—by two NiCp organometallic fragments does not change the structural arrangement of the cluster core and preserved the electronic picture.

Many boron-rich metallaboranes of the late transition metals mimic the boranes and obey the Wade-Mingos electron counting rules. But there are many exceptions, especially with early transition metals, which do not conform to these Wade-Mingos rules. In the history of metallaborane chemistry, compounds with low formal electron counts and anomalous structures have been reported since the beginning. Isocloso, hypocloso, etc. clusters were identified, where transition metal atoms generally show a tendency to occupy vertices of higher connectivity, 6 or more [17,18,19]. These structures are generally slightly hypoelectronic with only *n* rather than *n* + 1 seps [20]. Later, even more hypoelectronic species (*n* − 3 seps) with highly oblate (flattened) structures called oblatocloso deltahedra in contrast to spherical or prolate geometry [21], were discovered [22,23] and rationalized [24,25,26,27,28,29]. In these structures, the metal atoms are generally highly connected and occupy opposite vertices in such a way, that the M–M distance is consistent with the existence of a cross-cluster bond. DFT calculations suggested that these oblatocloso arrangements are indeed very stable due to an intricate mutual interaction of the bimetallic fragment and the borane cage [25].

To get more precise information about the shape and the electron count of these ‘rule-breakers’, theoretical computations are generally needed [1,12]. These calculations are useful to illustrate and discuss: (i) about the shape itself, (ii) which isomer is energetically preferred, (iii) about their bonding, electronic structure, electronic properties, etc. They are generally of the density functional theory (DFT) type, often complemented with a cornucopia of modern theoretical tools (quantum theory of atoms in molecules (QTAIM) analysis [30], Laplacian of the electron density [31], electron localization function (ELF) [32,33,34], natural bond orbital (NBO) analysis [35], etc.) which can provide deep insights into the nature of the chemical bonding of these anomalous molecules. Here, in this review, we illustrate the chemical bonding in some of the dimetallaborane compounds, especially those species having a core made of two metal atoms and a few boron atoms.

**Figure 1:**
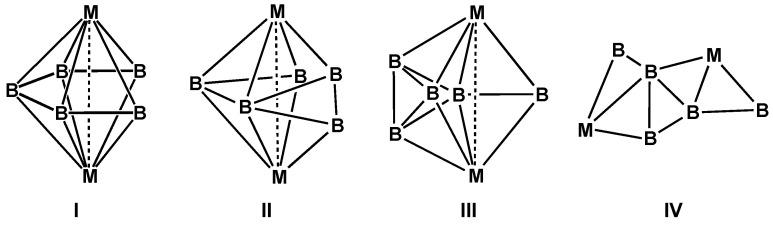
Different architectures reported for M_2_B_5_ metallaborane clusters.

During the 1990s, the chemistry of metallaborane clusters, which can be viewed as borane clusters with one or more vertices replaced by a transition metal fragment, observed a flourishing development [36,37,38,39,40]. Their chemical and physical properties make them promising candidates for use in different areas such as catalysis [41,42,43,44,45] or materials science etc. [46,47,48,49,50]. With the impetus of Fehlner who started to practice a fruitful synthetic method employing monoborane reagents and cyclopentadienyl metal chlorides [51,52], a large set of new metallaboranes have been characterized. Among them, many dimetallaboranes of early to late transition metals were synthesized and characterized with different size and structure. These dimetallaboranes mainly vary from five-vertex M_2_B_3_ clusters to seven-vertex M_2_B_5_ clusters and beyond [53,54,55,56,57]. The transition metals in the M_2_B_n_ clusters range from group 4 to group 9 with different skeletal electron donations. The structural arrangement of these clusters strongly varies according to their electron count. One of the important and largely explored cluster systems in metallaborane chemistry is the M_2_B_5_ clusters which can adopt different skeletal structures. The most largely encountered ones adopt a flattened hexagonal bipyramidal structure with a missing vertex and are hypoelectronic with only 6 seps (**I**, Figure 1). Due to their flattened and open structure, they are conveniently called oblatonido species with the two metal atoms on opposite sides in the flattened direction so that the metal–metal distances are short enough for direct metal–metal bonding interaction [25]. The examples of this kind of M_2_B_5_ species mostly range among the early transition metals such as group 5 in [(CpV)_2_B_5_H_11_] [58] and [(Cp*Ta)_2_B_5_H_11_] [59], group 6 in [(Cp*M)_2_B_5_H_9_] (M = Cr, Mo, W) [60,61,62,63,64,65], and group 7 in [(Cp*Re)_2_B_5_Cl_5_H_2_] [24] (Cp = C_5_H_5_, Cp* = C_5_Me_5_). Due to their unique structure, thorough theoretical studies were performed by us and others in order to get some insight into their electronic structure and chemical bonding [66,67,68,69,70]. Later, many heterometallic [63,71,72,73,74] and chalcogenated derivatives of oblatonido-M_2_B_5_ [75,76,77,78] were reported in the literature, which are isostructural as well as isoelectronic. In all these derivatives, one vertex in the parent cluster is replaced by an isolobal metal fragment or a chalcogen atom. Syntheses of most of these derivatives were obtained from the tendency of the unsaturated hypoelectronic clusters to react with metal or chalcogen fragments, which can readily donate further electrons to the cluster framework.

A few other structural arrangements with M_2_B_5_ composition are also known but far less common (**II**–**IV**, Figure 1). Their structures vary from 7-sep oblatoarachno to 9-sep open M_2_B_5_ structures. Types **II** [59,79,80] and **III** [81] adopt oblatoarachno geometries, which can be generated from the same parent structure. The number of representatives of these structural types is comparatively very limited. The last type of structure (**IV**, Figure 1) differs strongly from the others and is extremely rare, only known with group 4 metals, Zr and Hf [82,83]. In this review, we focus on the fundamental aspects, i.e., the synthesis, structure, electronic properties, and chemical bonding of these different structural types (**I**–**IV**) of M_2_B_5_ dimetallaborane clusters and their derivatives.

## 2. *Oblatonido*-M_2_B_5_ Clusters of Type I

### 2.1. Synthesis

**Scheme 1:**
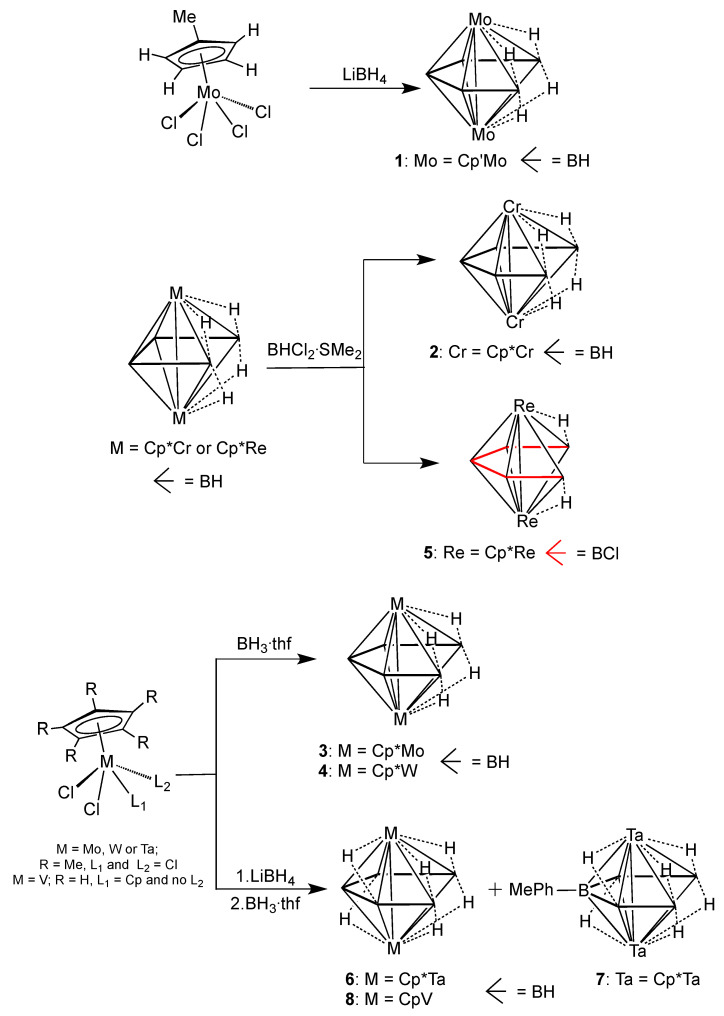
Synthesis of oblatonid*o*-M_2_B_5_ complexes **1**–**8** of type **I**.

Bullick et al. isolated the first M_2_B_5_ cluster [(Cp′Mo)_2_B_5_H_9_], **1**, (Cp′ = C_5_H_4_Me) in 1995 from the reaction of [Cp′MoCl_4_] with LiBH_4_ in diethyl ether [60]. Later, the Cp* analogs with Cr and Mo were reported by Fehlner and colls. from two different types of reaction [61]. [(Cp*Cr)_2_B_5_H_9_], **2**, was synthesized from the reaction of [(Cp*Cr)_2_B_4_H_8_] with BHCl_2_·SMe_2_ by addition of a BH unit. The synthesis of the orange crystalline solid [(Cp*Mo)_2_B_5_H_9_], **3**, was obtained from the reaction of [Cp*MoCl_2_]_2_ with LiEt_3_BH, followed by the treatment with BH_3_·thf (Scheme 1) [61]. These methods signify the high yield targeted synthesis of clusters of **2** and **3**. Interestingly, the reaction condition for the formation of **2** and **3** indicates that the addition of the fifth boron requires more forcing conditions in the case of chromium. On the contrary, a clean one-step synthesis occurs for Mo_2_B_5_ cluster **3**. The reaction of [Cp*MoCl_4_] or [Cp*MoCl_2_]_2_ with BH_3_·thf also led to the formation of **3** [62,63]. Somewhat later, **3** has also been synthesized and structurally characterized by Girolami’s group from the recation of [Cp*MoCl_2_]_2_ with [NaB_3_H_8_] [64]. Hence, it indicates **3** as a thermodynamically favorable product as it forms by any combination of the borane source with the molybdenum cyclopentadienyl precursor. The tungsten analog was reported a bit later, in 1999, in Fehlner’s group [65]. The reaction between [Cp*WCl_4_] or [Cp*WCl_2_]_2_ with six equivalents of BH_3_·thf affords to give [(Cp*W)_2_B_5_H_9_], **4**, through the [(Cp*WCl)_2_B_2_H_6_] intermediate. In 2004, another member of this series, namely [(Cp*Re)_2_B_5_Cl_5_H_2_], **5**, was reported by the same group from a typical reaction of [(Cp*Re)_2_B_4_H_8_] with an excess of BHCl_2_·SMe_2_ [24]. This compound represents the first example of a dimetallaborane with fully chlorinated boron atoms. The synthesis of **5** is very similar to the synthesis of **2** with borane addition. Later, we reported the Ta analog [(Cp*Ta)_2_B_5_H_11_], **6** [59]. This compound was synthesized from the reaction of [Cp*TaCl_4_] with a six-fold excess of LiBH_4_ followed by BH_3_·thf in toluene, along with a few other tantalaboranes which include the substituted analog, [(Cp*Ta)_2_B_5_H_10_(C_6_H_4_CH_3_)], **7** [59]. Apart from **7**, a few more substituted derivatives of **6** are known from the C-H activation of arenes and heteroarenes by **6** (these compounds are not described in this review) [84]. The last entry in this series is [(CpV)_2_B_5_H_11_], **8**, which was synthesized from a similar reaction as that of Ta system (Scheme 1) [58].

### 2.2. Structure and Electron Count

**Figure 2:**
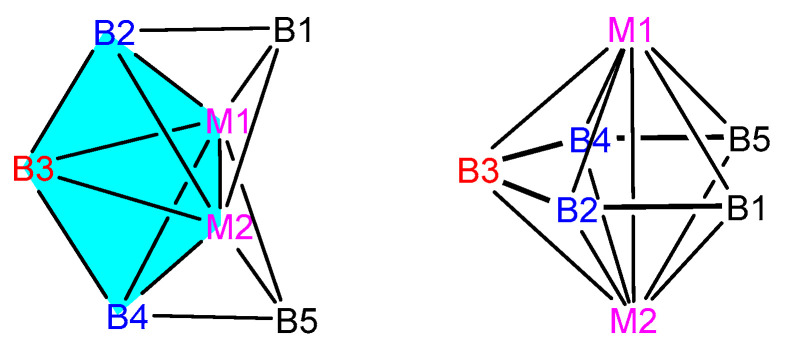
Two different views of the cluster cage of M_2_B_5_ clusters of type **I**.

The molecular structure of clusters **1**–**8** was confirmed by single-crystal X-ray diffraction studies. The structure of these clusters was first considered as a bicapped closo-trigonal bipyramid where the M1-M2-B3 triangle is the trigonal plane, B2 and B4 are the apical atoms and two M-M-B planes (M1-M2-B2 and M1-M2-B4) are capped by B1 and B5 atoms (Figure 2). The core of these molecules is rather highly symmetric (C_2_*_v_* symmetry). Thus, the metal atoms as well as B1 and B5, and B2 and B4 are symmetry related (symmetry-related atoms are labeled with the same color in Figure 2). Accordingly, experimental ^11^B NMR spectra of **1**–**8** exhibit three different types of boron resonances in a 2:2:1 ratio (Table 1). According to this structural description, an M–M bond is assumed in all of these clusters in agreement with the M–M bond distances measured experimentally (Table 1). Though, the cluster structure of **1**–**8** is similar, the number and position of the bridging hydrogen atoms varies in different metal analogs to reach their electron count requirement. The differences in ^1^H chemical shifts of the M-H-B protons in **1**–**8** are negligible, but there is a significant difference in the ^11^B chemical shifts of the three types of boron atoms in the M_2_B_5_ core on moving from first- to third-row transition metals (Table 1).

As stated earlier, bicapped closo-trigonal bipyramidal species **1**–**8** are characterized by 6 seps or 46 cves. This sep count is expected considering that, according to the capping principle which states that capping a face should not generally modify the favored number of seps [85,86]. For example, a *m*-capped-*n*-vertex closo structure (*m* = 2, *n* = 7) will have (*n* − *m*) + 1, i.e., 6 skeletal electron pairs. The observed 46-cve count is theoretically obtained taking into account the principle of polyhedral condensation (or fusion) which stipulates that the total cve count of fused clusters is equal to the sum of the cve of the parent polyhedra minus the cve of the shared unit (atom, pair of atoms, triangle, etc.) [8,9,10,87]. Thus, the bicapped closo-trigonal bipyramidal clusters **1**–**8** (Figure 2) can then be viewed as the fusion of an M_2_B_3_ closo-trigonal bipyramid (42 cves) with two M_2_B_2_ tetrahedra (2 × 40) through two M_2_B triangular faces (–{2 × 38}). This results to a cve count of 46 (42 + 80 − 76), equal to the cve for the corresponding molecular formula.

**Figure 3:**
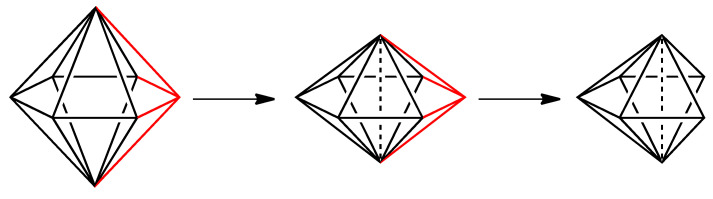
Generation of the oblatonido-deltahedral cage of M_2_B_5_ clusters **1**–**8**.

Alternatively, the structure of these species can also be described as a derivative of a triple-decker sandwich structure in which the middle deck is made by the B_5_ plane and the Cp* ligands act as outer decks, somewhat flattened along the M–M axis [25]. In other words, clusters **1**–**8** adopt a structure which can be derived from a (flattened) closo-hexagonal bipyramid by removal of an equatorial vertex (Figure 3). These species identified as oblatonido metallaboranes are considered as hypoelectronic compounds with only 6, i.e., *n* − 1 seps instead of 9 (*n* + 2) seps which would be expected according to the Wade–Mingos rules.

### 2.3. Electronic Structure

These dimetallaboranes were theoretically studied much earlier to get a better understanding about their chemical bonding. Semi-empirical Fenske–Hall molecular orbital calculations were first performed on Cp analogs (**2’** and **3’**) which used to tentatively analyze the electronic factors underlying in the short M–M bond and other different structural features in clusters **2** and **3** [63]. A comparison was made with the Cp analog of the reactive starting precursor, [(Cp*Cr)_2_B_4_H_8_]. Results indicated that, as expected, cluster **2’** was more thermodynamically stable (larger energy gap between the highest occupied molecular orbital (HOMO) and the lowest unoccupied molecular orbital (LUMO) and showed stronger Cr–Cr bonding interaction (larger Mulliken overlap population) than in the starting species.

Subsequently, a detailed study was performed at the DFT level of theory on the rhenium species [(Cp*Re)_2_B_5_Cl_5_H_2_], **5** [24]. The primary aim was to locate the Re−*H*−B bridging hydrogens more precisely which were identified by NMR spectroscopy but not by X-ray crystallography. Two possible isomers corresponding to *cis* or *trans* arrangements of the two bridging hydrogens on the open M1B1M2B5 face (Figure 2) were considered and computed. Similar Re–Re distances were computed in both isomers, comparable to that experimentally measured (2.7641(3) Å). Computed Re−H and B−H bond lengths marginally differ from one isomer to the other. Moreover, the two isomers are nearly isoenergetic. This is consistent with fluxional behavior of **5** observed in solution. The calculated NMR chemical shifts were also very similar, but values of the *trans*-isomer were closer to the experimental values.

Later, King and coworkers studied various possible geometries for [(CpM)_2_B_5_H_9_] clusters with early and late transition metals of the second and third rows including the experimentally known species **2** and **3** [68]. Different structures were predicted for each metal, depending upon their electronic requirement, which in most cases, could be related to the Wade–Mingos electron counting rules. Gratifyingly, the bicapped trigonal bipyramid structure of **2’** and **3’** (Cp analogs of **2** and **3**) were found largely thermodynamically more stable than all other possible geometries, consistent with the experimental observation [68].

At the same time, the structural, electronic, and NMR properties of some dimetallaboranes with varied stoichiometry and structure, including the group 6 oblatonido-clusters **2**–**4**, were studied to test the strength of different DFT methods in analyzing their properties [69]. In clusters **2**–**4**, computations indicated that the ^11^B NMR chemical shifts of the boron atoms attached directly to the metal atoms (B2, B4, and B3, Figure 2) show a large systematic shift to higher field, whereas the chemical shifts at upper field for the boron atoms connected to the metal atoms via M−H−B bridged bonds (B1 and B5, Figure 2) hardly deviate from Cr to Mo to W species. The nature of the M–M interaction in clusters **2**–**6** and **8** was further probed using Cp-analog models employing the electron density and its derivatives within the framework of the QTAIM method and the aid of bonding indicators [70]. Results revealed that both through-space and through-bond (via boron atoms of the ring) interactions account for a substantial metal–metal interaction with a covalent bond order around 1.

## 3. Heterometallic Derivatives of *Oblatonido*-M_2_B_5_ Clusters

### 3.1. Synthesis

**Scheme 2:**
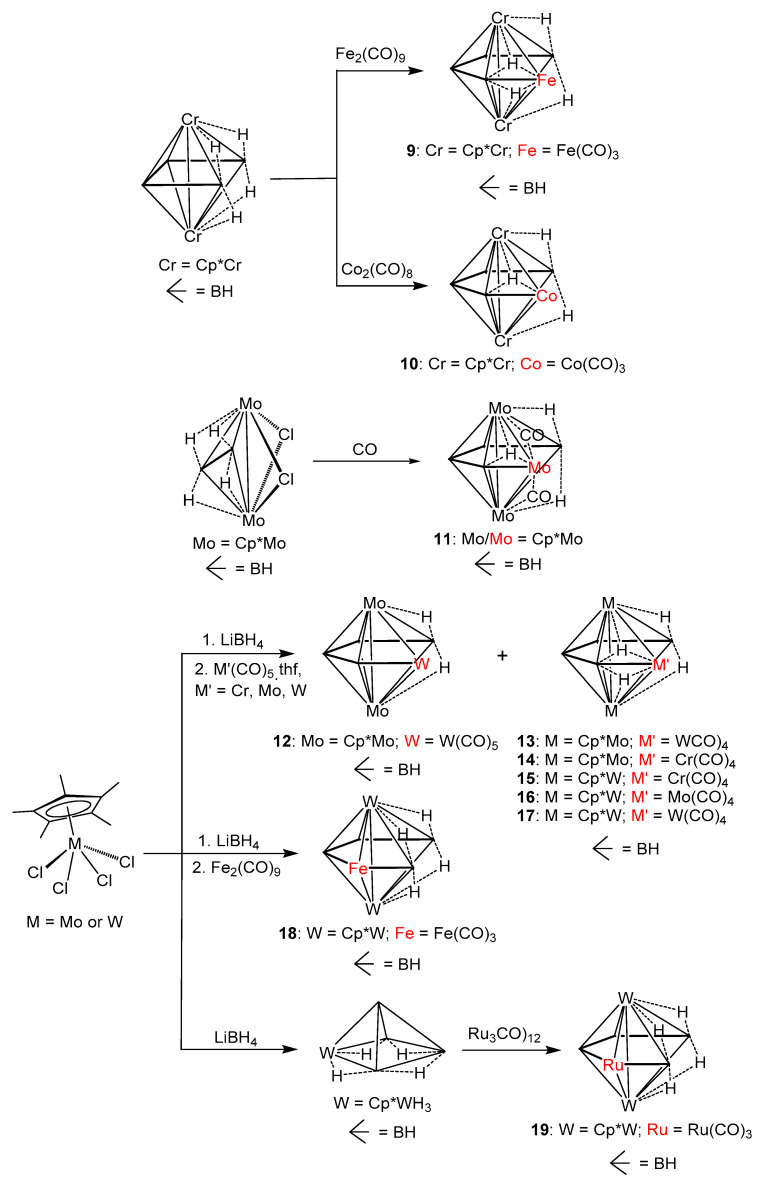
Synthesis of heterometallic oblatonido-M_2_M’B_4_ derivatives **9**–**19**.

The isolobal concept mentioned earlier is of value in theoretically generating new mixed main-group–transition-metal clusters [1]. In other words new species where one or more *p*-block boron fragments are substituted by *d*-block metal moieties in metallaborane compounds must be possible. Indeed, the first metalla-derivative of oblatonido-M_2_B_5_ clusters, [(Cp*Cr)_2_B_4_H_8_Fe(CO)_3_], **9** was reported by Fehlner and colls. from the room temperature reaction of the unsaturated cluster [(Cp*Cr)_2_B_4_H_8_] with Fe_2_(CO)_9_ [71]. Similarly, they showed later that the reaction with Co_2_(CO)_8_ led to the mixed-metal metallaborane [(Cp*Cr)_2_B_4_H_7_Co(CO)_3_], **10**, (Scheme 2) [63]. In both cases the [(Cp*Cr)_2_B_4_H_8_] moiety behaves as a ligand which coordinates to the metal fragment. Recently, we described the synthesis of [(Cp*Mo)_3_B_4_H_7_(µ-CO)_2_], **11**, in which one of the {BH} vertices in **3** has been replaced by a Cp*Mo unit, from the reaction of [(Cp*Mo)_2_(μ-Cl)_2_B_2_H_6_] at room temperature with CO gas [72]. A series of Mo and W derivative compounds with different metal carbonyl fragments were obtained previously from the reaction of the intermediate formed from the reaction of [Cp*MCl_4_] (M = Mo or W) and LiBH_4_, with the corresponding metal carbonyls M(CO)_5_·thf (M = Cr, Mo, W). The reaction of the Mo metallaborane intermediate yielded [(Cp*Mo)_2_B_4_H_6_W(CO)_5_], **12**, [(Cp*Mo)_2_B_4_H_8_W(CO)_4_], **13**, and [(Cp*Mo)_2_B_4_H_8_Cr(CO)_4_], **14** [72,73]. In the case of W system, the similar reaction yielded [(Cp*W)_2_B_4_H_8_M’(CO)_4_], **15–17** (**15**: M’ = Cr; **16**: M’ = Mo; **17**: M’ = W) [73]. Finally, reaction of the W intermediate with Fe_2_(CO)_9_ generated [(Cp*W)_2_B_4_H_8_Fe(CO)_3_], **18** [73], while the reaction of the isolated intermediate [Cp*WH_3_(B_4_H_8_)] with Ru_3_(CO)_12_ resulted in the analogous cluster, **19** (Scheme 2) [74].

### 3.2. Structure and Electron Count.

Molecules M_2_M’B_4_ **9**–**19** have a similar oblatonido-hexagonal bipyramidal core to that of the parent M_2_B_5_ clusters in which one {BH} unit has been substituted by isolobal metal fragments. In species **9**–**17**, M’ occupies one of the remote sites of the M’B_4_ open ring. On the other hand, M’ is found inside the open ring in compounds **18** and **19** (Scheme 2). M−M, M−B, and B−B distances in these complexes are comparable overall with the corresponding ones in the parent compounds **2**–**4** (compare Table 1 and Table 2). All possess 6 seps as their M_2_B_5_ parents, or 56 cves. Depending upon the nature of M’, the number and the connectivity (μ or μ_3_) of the bridging hydrogen atoms varies from two to four to adjust the expected 6-sep count. Due to the lowering of symmetry of these molecules with respect to their M_2_B_5_ congeners, NMR measurements indicate that most of them show four (1:1:1:1 ratio) and in a few cases three (2:1:1 ratio) ^11^B resonances (Table 2), and two types of M−H−B bridging hydrogen atoms (μ or μ_3_).

### 3.3. Electronic Structure

**Figure 4:**
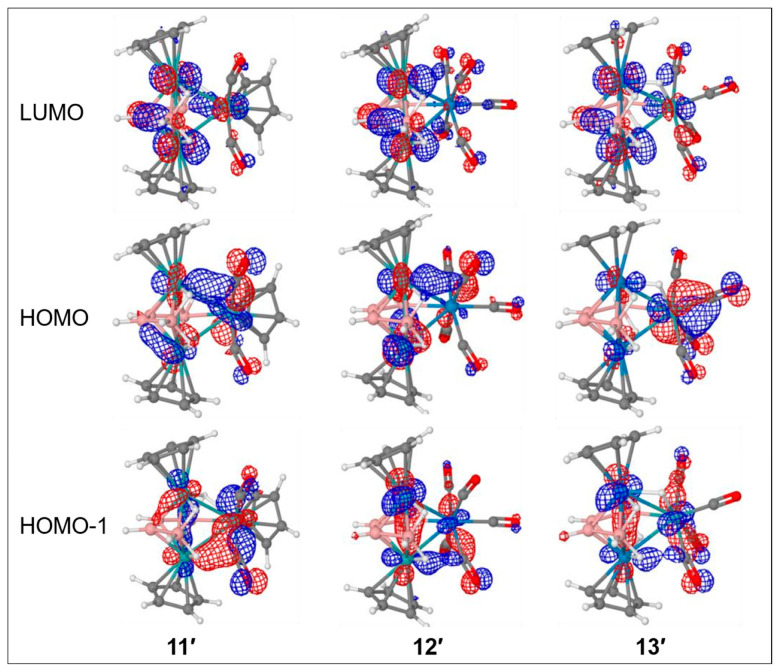
HOMO, HOMO-1 and LUMO plots of **11’**, **12’**, and **13’**. Reprinted and adapted with permission from Ref. [72]. Copyright (2019) American Chemical Society.

Most of these M_2_M’B_4_ species were theoretically studied. A fragment analysis employing Fenske–Hall molecular orbital calculations was first carried out on Cp analogs of **9** [71] and **10** [63] to show that strong bonding interactions are occurring between the M’ organometallic fragment and the rest of the molecule. Later, a DFT computational analysis was performed on models **11’**–**13’**, Cp analogs of **11**–**13** [72]. A comparison of **11’** with its M_2_B_5_ parent **3** indicated that the replacement of a {BH_2_} vertex in the open face of **3** by a {Cp(Mo(CO)_2_} unit in **11’** slightly stabilizes the LUMO and destabilizes the HOMO, resulting in a reduced HOMO-LUMO energy gap. A qualitative MO analysis shows that both the HOMO and HOMO-1 mostly account for a strong (Cp)Mo–Mo(Cp) bonding character (Figure 4), similar to that in the parent cluster **3**. This strong Mo–Mo interaction is supported by an NBO analysis which shows a Mo_apical_–Mo_apical_ Wiberg bond index (WBI) [88,89] 1.5 times higher than the corresponding Mo_apical_–Mo_equatorial_ ones [72]. Though **12’** slightly differs from **11’** due to the absence of bridging hydrogen interaction with the W(CO)_5_ fragment, a molecular orbital analysis shows very similar bonding properties [72]. HOMO, HOMO-1, and LUMO comparable to those in **11’** are computed for **12’** and **13’** (Figure 4). The HOMO and HOMO-1 show an important σ-bonding character between the metal atoms while the LUMO shows some δ-bonding character. In **12’**, an acceptor-donor interaction between the W(CO)_5_ metal fragment and the rest of the cluster is reflected by a NBO charge analysis which shows a rather high negative charge on W (−1.57) with respect to Mo atoms (−0.87 and −0.84).

**Figure 5:**
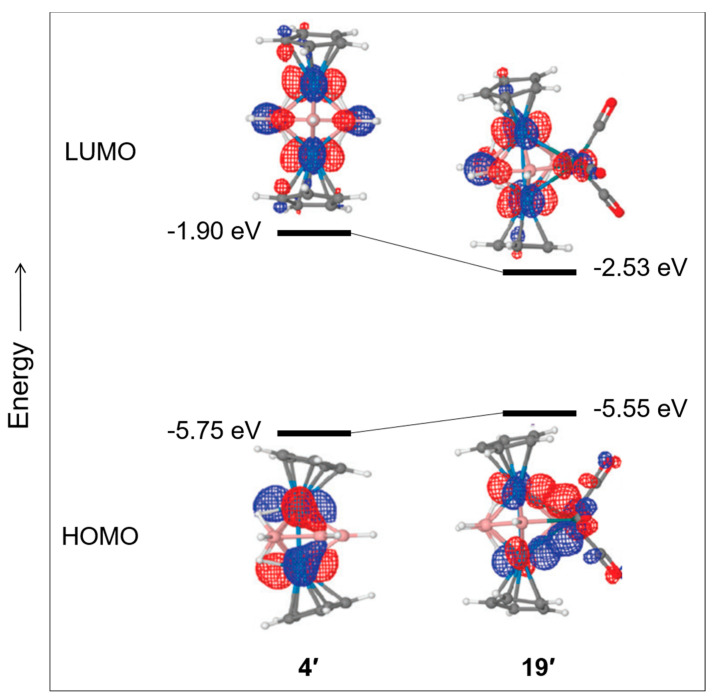
HOMO and LUMO plots of **4’** and **19’** Adapted from Ref. [74].

Theoretical calculations were also performed on **19’**, the Cp analog of **19** [74]. An MO analysis showed that this time, the HOMO is mainly localized on the equatorial Ru metal atom and to a lesser extent on the apical W atoms with a strong Ru−W σ-bonding character and some W-W δ* character (Figure 5). The LUMO possesses some W−W δ-bonding character. W−W δ* and δ character for the HOMO and LUMO, respectively, was also observed for the parent molecule **4’** (Figure 5).

## 4. Chalcogen Derivatives of *Oblatonido*-M_2_B_5_ Clusters

### 4.1. Synthesis

**Scheme 3:**
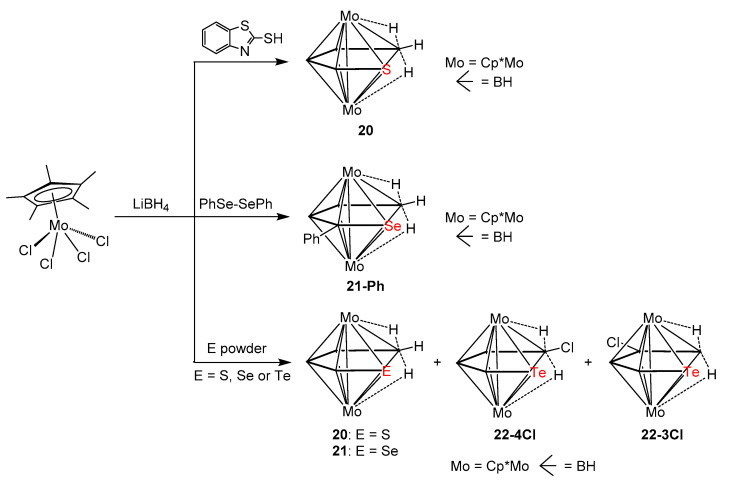
Synthesis of chalcogen M_2_EB_4_ derivatives **20**–**22**.

Similarly to the heterometallic M_2_M’B_4_ derivatives, a few chalcogen derivatives have also been synthesized in our laboratory where a {BH} unit is replaced by a chalcogen E atom (E = S, Se and Te). These M_2_EB_4_ derivatives are only reported with Mo as the metal. Many methods were employed so far for the synthesis of these compounds (Scheme 3). The sulfur derivative, **20**, was first synthesized from the reaction of the in situ intermediate generated from the Cp*MoCl_4_ and LiBH_4_ reaction with the 2-mercaptobenzothiazol [76]. The Se analog, **21-Ph**, was synthesized from a molybdaborane intermediate reacting with the Ph_2_Se_2_ reagent [77]. In this structure, one of the terminal hydrogen atoms on boron is substituted by a phenyl group. Finally, the Te-derivatives **22-4Cl** and **22-3Cl** were synthesized from a similar reaction with Te powder [75,78]. The same method with other chalcogen powders yielded the S and Se analogs as well (Scheme 3) [78].

### 4.2. Structure and Electron Count

The structure of all analogs **20**–**22** is isostructural to the parent molecule **3**, with one of the BH_3_ units on the open face replaced by an isolobal chalcogen atom. Hence, these species can also be viewed as oblatonido-Mo_2_EB_4_ clusters with electron counts of 6 seps or 46 cves as the parent Mo_2_B_5_ species **3**. Compound **22-3Cl** was only characterized spectroscopically. All the others were characterized crystalographically also. The Mo-Mo, average Mo-B, and B-B distances in these clusters (Table 3) are comparable with those measured for **3**. In more detail, the Mo-Mo distance in the Te analog **22-4Cl** is slightly elongated but slightly shortened in the S and Se analogs **20** and **21**. All of these complexes show four ^11^B NMR resonances corresponding to the four unique boron atoms in 1:1:1:1 ratio (Table 3). The most up-field chemical shift is assigned to the boron atom attached to the chalcogen atom. The value increases upfield in the order Te < Se < S. The other boron atoms are highly deshielded.

### 4.3. Electronic Structure

**Figure 6:**
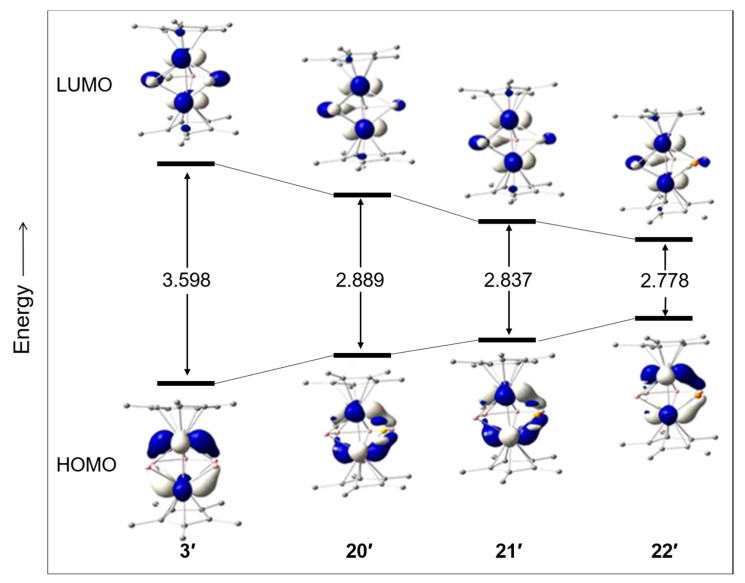
HOMO and LUMO plots of **3’** and **20’**–**22’** (HOMO-LUMO gaps in eV). Reprinted and adapted with permission from Ref. [78]. Copyright (2013) American Chemical Society.

DFT calculations were carried out to analyze the electronic structure of the Cp-analog models **20’**–**22’** [66,78,90]. The molecular orbital study shows a significant destabilization of their HOMO with respect to that of model **3’** (Cp analog of **3**) (Figure 6), that suggests a higher reactivity as experimentally observed. In addition, moving from S to Te, there is a gradual decrease in the HOMO-LUMO energy gap. The destabilization of the HOMO is attributed to the introduction of the π-donor chalcogen atoms into the cluster cages.

## 5. *Oblatoarachno*-M_2_B_5_ Clusters of Type II and III

### 5.1. Synthesis

**Scheme 4:**
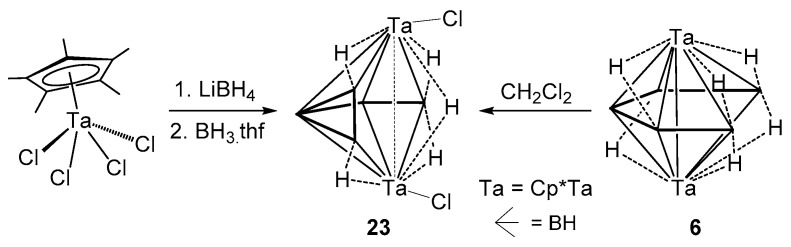
Synthesis of compound **23** from different methods.

As detailed in the beginning, some metallaboranes of M_2_B_5_ composition adopt geometrical arrangements which differ from that of type **I**. The first oblatoarachno-M_2_B_5_ compound of type **II** (Figure 1), [(Cp*TaCl)_2_B_5_H_11_], **23**, was synthesized by our group from the same reaction which yielded clusters **6** and **7** (Scheme 4) [59]. Later, compound **23** and its other halogenated derivatives, **24** and **25**, were synthesized by the halogenation of [(Cp*Ta)_2_B_5_H_11_], **6**, with CH_2_Cl_2_, CH_2_Br_2_, and I_2_ [79]. The monochlorinated derivative [(Cp*TaCl)_2_B_5_H_10_Cl], **26**, was also isolated from similar reaction conditions [80].

**Scheme 5:**
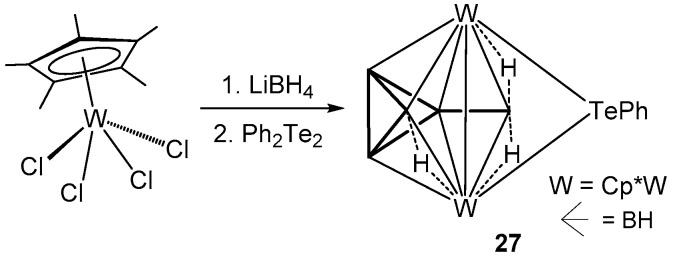
Synthesis of compound **27**.

Recently, another type of M_2_B_5_ oblatoarachno species of type **III** (Figure 1), [(Cp*W)_2_(TePh)B_5_H_5_(*µ*-H)_3_], **27**, was reported, synthesized from the reaction of a W-metallaborane intermediate with Ph_2_Te_2_ (Scheme 5) [81]. Though this synthetic method is similar to the synthesis of **21-Ph**, here, the reaction yielded a new structural type rather than forming a derivative cluster.

### 5.2. Structure and Electron Count

**Figure 7:**
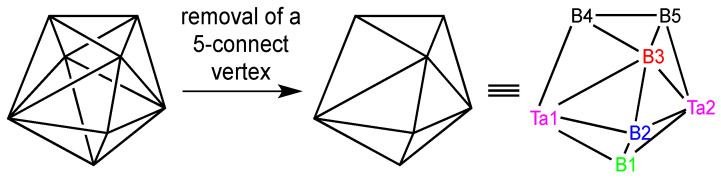
Generation of the cluster structure of cluster **23**.

Clusters **23**–**26** were characterized by X-ray crystallography. The average B-B distance is marginally shorter, whereas average Ta-B distances are longer in **23**–**26** (Table 4) than the corresponding ones in **6**. The Ta−Ta distance in **23**–**26** (ca. 3.23 Å) is substantially longer than that in **6** (2.93 Å). This is too long for a single M−M bond but also too short to have no interaction between the two metal centers. The ^11^B NMR spectroscopy of **23**–**25** shows four resonances in a 2:1:1:1 ratio as the boron atoms B4 and B5 are symmetry related (Figure 7). In **26**, due to the lowering of symmetry, each boron atom becomes unique, and five different chemical shifts are observed.

**Figure 8:**
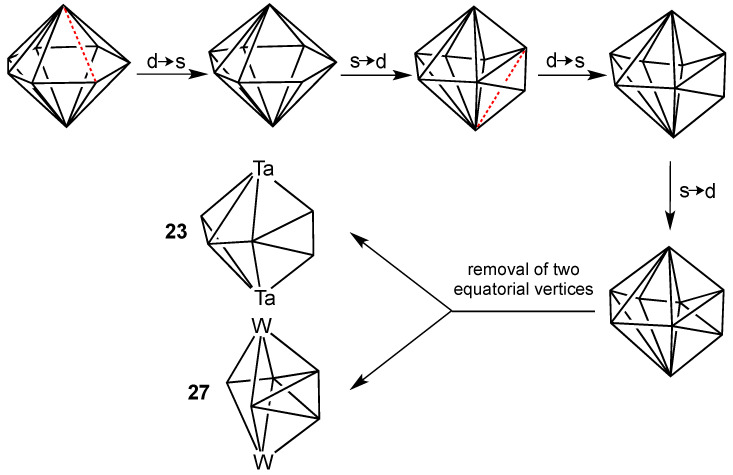
Generation of the oblatoarachno structure of clusters **23** and **27** from an oblatocloso structure.

The core structure for clusters **23**–**26** was initially considered as a nido-structure derived from a closo-dodecahedron as shown in Figure 7 [59]. But this is not in agreement with the usual Wade–Mingos rules since these clusters possess only 7 seps ([2 × (−1) (Cp*TaX) + 5 × 2 (BH) + 6 × 1 (bridging H)]/2). Alternatively, these clusters may be seen as 7-vertex oblatoarachno-M_2_B_5_ species, the structure of which can be derived by the removal of two equatorial boron atoms from a 9-vertex oblatocloso-M_2_B_7_ cluster (Figure 8. This makes these clusters hypoelectronic, with fewer valence electrons than generally observed for a canonical arachno-structure of the same nuclearity. But according to a specific electron counting rule proposed by King [25], *n*-vertex oblatoarachno-clusters should have a non-Wadean electron count of *n* skeletal electron pairs which matches well with the 7-sep count of **23**–**26**.

**Figure 9:**
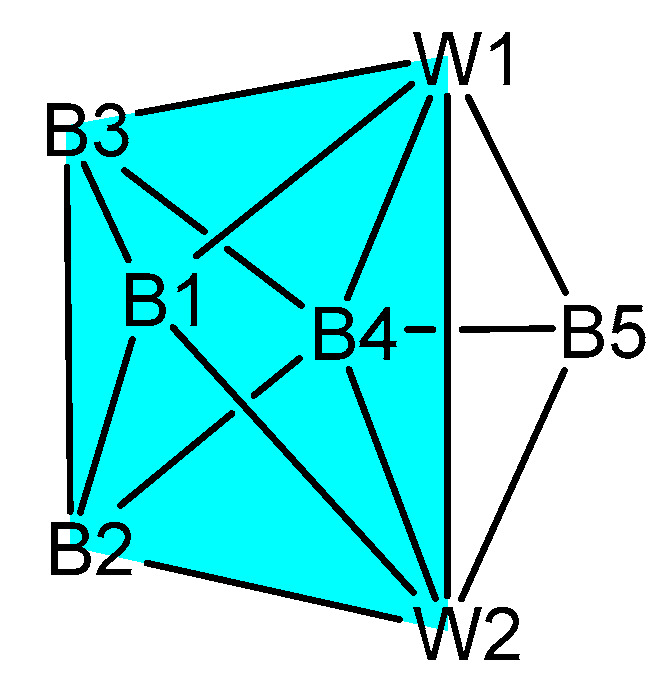
Core structure of cluster **27**.

In **27**, the W−W bond length (2.85 Å) bridged by a TePh group is slightly longer than in **4** (2.81 Å) [65]. Its structure can also be viewed in different ways. With a rather short W−W bond length, it can be first considered as a capped octahedron in which the W_2_B_4_ octahedron (W1, W2, B2, and B3 as the base, and B1 and B4 as the apical vertices) is capped on the W1-W2-B4 face by a BH_3_ unit (B5, Figure 9), similar to the core structure found for [(Cp*M)_3_B_4_H_4_] (M = Co, Rh, and Ir) [41,91,92]. In other words, the structure of **27** results from the fusion of an octahedron with a tetrahedron through a triangular face. According to the condensation principle [8,9,10,87], a cve count of 48 is expected (46 ([W_2_B_4_] octahedron) + 40 ([W_2_B_2_] tetrahedron) − 38 ([W_2_B] triangle) = 48 e). This is indeed the observed cve count of **27** (2 × 11 (Cp*W) + 5 × 4 (BH) + 3 × 1 (µ-H) + 1 × 3 (µ-TePh) = 48).

Alternatively, **27** can also be considered as 7-sep hypoelectronic cluster [25] having an M_2_B_5_ oblatoarachno-structure which can be derived from a 9-vertex closo heptagonal bipyramid with two diamond-square-diamond (dsd) rearrangements and removal of two vertices (Figure 9). Interestingly, the core structure of **23** and its derivatives **24**–**26**, and that of **27** can be seen as structural isomeric forms (Figure 8).

### 5.3. Electronic Structure

The electronic structure of all the halogenated Ta-species **23**–**25** was extensively studied [79]. Pertinent results are reported in Table 5. The HOMO−LUMO gaps of **23**–**25** are around 2 eV which is slightly smaller than that for the parent cluster **6** which is 2.3 eV. The energy trend is in accord with the electronegativity of the halogens (Cl > Br >I).

**Figure 10:**
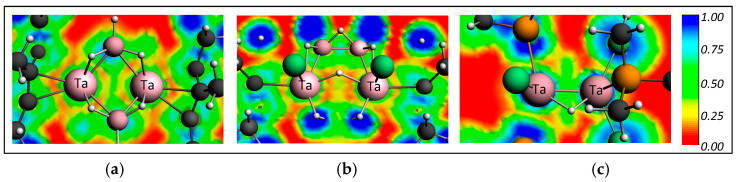
DFT ELF plots for clusters **6** (in the Ta-C(Cp)-Ta plane, (**a**), **23** (in the Ta-B-Ta plane, (**b**), and [Ta_2_(PMe_3_)_4_Cl_4_(μ-H)_2_] (in the Ta-Cl-Ta plane, (**c**). ELF values vary from 0 to 1, the upper limit corresponding to perfect electron-pair localization (important covalent character), whereas the reference value of 0.5 corresponds to a perfect delocalization (homogeneous electron gas). Reprinted and adapted with permission from Ref. [79]. Copyright (2012) American Chemical Society.

Some of the information about the nature of the bonding between the Ta atoms in clusters **23**–**25** was obtained by an analysis of the electron localization function (ELF) [32,33,34]. ELF is directly related to the electron pair probability density, and its graphical representation contributes to the understanding of electron localization/delocalization and consequently to the degree of metal−metal interaction. Two-dimensional electron density distribution plots are depicted in Figure 10 for **23**, as well as for the parent compound **6** (*d*[Ta−Ta] = 2.926 Å) and [Ta_2_(PMe_3_)_4_Cl_4_(μ-H)_2_] [93], where a short Ta−Ta bond length is observed (2.545 Å), for comparison. A high delocalization of electrons between the metals is observed in **23** and **6**, as generally computed for complexes with weak M−M interactions [94]. As expected, a much more important electron localization is computed for [Ta_2_(PMe_3_)_4_Cl_4_(μ-H)_2_]. The weak Ta−Ta interaction in **23** was also probed by the DFT-computed bond multiplicity indices (BMI) based on the Nalewajski–Mrozek method [95]. Ta−Ta BMI values in **23**–**25** are 0.23, much weaker than the values computed for **6** (0.58) and for [Ta_2_(PMe_3_)_4_Cl_4_(μ-H)_2_] (1.80), corroborating well with experimental observations.

DFT calculations were also performed on **27’**, the Cp analog of **27** [81]. A HOMO-LUMO energy gap smaller than that of the parent cluster **4’** was computed. The computed WBI values indicate strong W−W and W−Te bonding interactions. Finally, the topology analysis of the Laplacian, −∇^2^*ρ*(r), of the total electron density, identifies 3c−2e bonds in the B1−B2−B3 and B2−B3−B4 triangles of the cluster cage (see Figure 9 for atom numbering).

## 6. Open M_2_B_5_ Clusters of Type IV

### 6.1. Synthesis

**Scheme 6:**
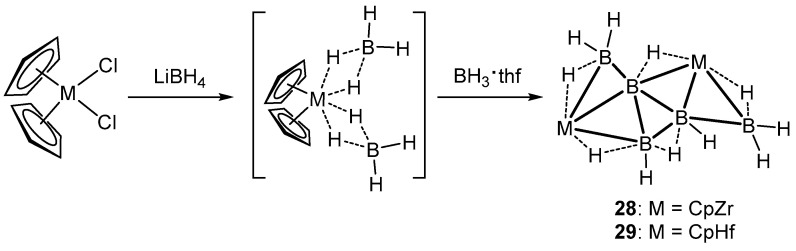
Synthesis of the complexes [(Cp_2_M)_2_B_5_H_11_] (M = Zr, Hf), **28** and **29**.

The last M_2_B_5_ structural type **IV** (Figure 1) is encountered only for two representatives so far. The first example, [(Cp_2_Zr)_2_B_5_H_11_], **28**, was synthesized in 2015 from our group through the thermolysis of [(Cp_2_Zr)(BH_4_)_2_], generated from the fast metathesis reaction of [Cp_2_ZrCl_2_] and LiBH_4_·thf with BH_3_·thf [82]. Cluster **28** was the first example of a homometallic neutral zirconaborane. Later, in 2018, the hafnium analog, **29** was synthesized in a similar manner with a slight modification of the reaction conditions (Scheme 6) [83].

### 6.2. Structure and Electron Count

**Figure 11:**
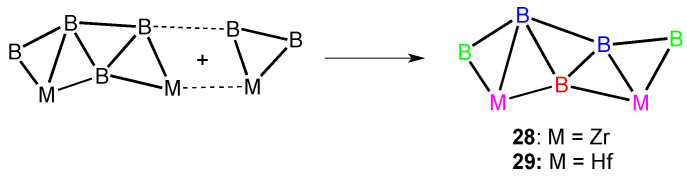
Generation of the core structure of compounds **28** and **29** from cluster fusion.

The structures of **28** and **29**, obtained from X-ray crystallography, show a highly open architecture with no metal−metal interaction in contrast to **I**–**III** (Figure 1). The arrangement of atoms in **28** and **29** resembles that of a substituted cyclopropane molecule with a central B_3_ ring. This B_3_ ring is similar to the [B_3_H_8_]^−^ structural matrix encountered in many borane compounds [96,97,98,99,100,101,102]. Pertinent bond length parameters are given in Table 6. ^11^B NMR spectroscopy shows three boron chemical shifts with a 2:2:1 ratio. Similar boron atoms are marked in the same color in Figure 11 (right). **28** and **29** possess 9 seps ([2 × 2 (Cp_2_M) + 1 × 3 (B) + 2 × 2 (BH) + 2 × 1 (BH_2_) + 5 × 1 (µ-H) = 18]/2) but their structure cannot be generated from a single structure. It can be generated however, from different ways such as the fusion of a six-vertex arachno M_2_B_4_ fragment analogous to B_6_H_12_, with an MB_2_ triangle, as shown in Figure 11.

As shown in Figure 11, the structure of **28** and **29** can result from the fusion of an arachno M_2_B_4_ moiety with an MB_2_ triangle through an MB edge, leading to an expected cve count of 54 (50 (arachno M_2_B_4_) + 28 (MB_2_ triangle) − 24 (MB edge) = 54). This is the actual cve count of **28** and **29** (2 × 14 (Cp_2_M) + 1 × 3 (B) + 2 × 4 (BH) + 2 × 5 (BH_2_) + 5 × 1 (µ-H) = 54).

### 6.3. Electronic Structure

**Figure 12:**
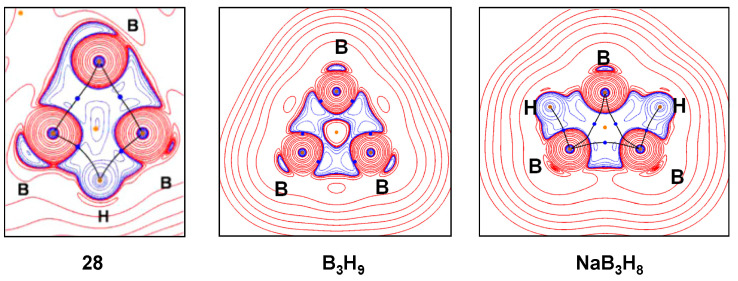
Laplacian contour map (–∇^2^*ρ*) of the electron density in the B_3_ plane of **28** (left), B_3_H_9_ (middle), and NaB_3_H_8_. (right). Red and blue contour lines indicate areas of charge concentration (∇^2^*ρ* < 0) and areas of charge depletion (∇^2^*ρ* > 0), respectively. Reprinted and adapted with permission from Ref. [82]. Copyright (2015) American Chemical Society.

The electronic structure of **28** was elucidated [82]. The optimized structure and the computed ^11^B NMR chemical shifts are in a very good agreement with the experimental data. Interestingly, an NBO analysis indicated a close relationship of the central B_3_ ring of **28** and the borane analogs NaB_3_H_8_ and B_3_H_9_. The topological analysis of the Laplacian of the electron density also shows similar topological features for these three molecules, as shown in Figure 12.

## 7. Conclusions

Fehlner pioneered the development of metallaborane chemistry a few decades ago. Subsequently, we and others made a large number of metallaborane clusters and their derivatives with various structural arrangements. Among them, M_2_B_5_ clusters and derivatives constitute an important class of those compounds. The transition metals present in these M_2_B_5_ species span from group 4 to group 7, particularly with group 6 elements. Their structure varies from oblatonido-, oblatoarachno-, to arachno-type open structures. The most open cluster architecture is observed for a couple of examples with group 4 early-transition metals. Closer M_2_B_5_ geometries such as oblatonido and oblatoarachno structures with M−M interactions dominate with group 5 and 6 metals. For group 7 metals, only one oblatonido-M_2_B_5_ structure is known with Re. Many of these species appear to be hypoelectronic clusters and are often considered as ‘rule breakers’ with respect to the classical Wade–Mingos electron rules. This is due to their unique highly oblate (flattened) deltahedral structures featuring a cross-cluster M−M interaction. Many theoretical calculations were performed to elucidate their electronic structure and chemical bonding properties. More specifically, a lot of theoretical works were devoted to the understanding of the nature of the cross-cluster M−M interaction in these species.

In this review, we have focused on the fundamental aspects, i.e., the synthesis, structure, electronic properties, and chemical bonding of the transition metal M_2_B_5_ clusters known in the literature. Examples which have been discussed have illustrated how, in synergy with experiments, computational results can provide additional valuable information to provide further insights about the electronic properties and electronic requirements which govern their architecture and thermodynamic stability. The field of metallaborane chemistry still remains a fertile ground of investigation that continues to create interest not only for experimentalists, but also among theoriticians, concerning viable predictions, particularly the synthesis of new metallaborane clusters of diverse stoichiometry with late-transition metals or mixed early- and late-transition metals.

## Figures and Tables

**Table 1:** Skeletal electron pair (sep), selected structural parameters and ^11^B chemical shifts of **1**–**8**.

Metallaborane	sep	*d* [M-M] (Å)	*d*_avg_ [M-B] (Å)	*d*_avg_ [B-B] (Å)	^11^B NMR (ppm)
[(C_5_H_4_MeMo)_2_B_5_H_9_] (**1**)	6	2.81	2.26	1.73	61.6, 59.7, 27.8
[(Cp*Cr)_2_B_5_H_9_] (**2**)	6	2.62	2.15	1.67	91.5, 86.2, 25.0
[(Cp*Mo)_2_B_5_H_9_] (**3**)	6	2.81	2.25	1.72	62.9 (3 B), 25.8
[(Cp*W)_2_B_5_H_9_] (**4**)	6	2.82	2.24	1.71	49.2, 46.9, 26.8
[(Cp*Re)_2_B_5_Cl_5_H_2_] (**5**)	6	2.76	2.20	1.75	88.3, 48.3, 28.1
[(Cp*Ta)_2_B_5_H_11_] (**6**)	6	2.93	2.33	1.80	44.8, 23.9, 3.7
[(Cp*Ta)_2_B_5_H_10_(C_6_H_4_CH_3_)] (**7**)	6	2.91	2.33	1.82	51.3, 20.8, 4.1
[(CpV)_2_B_5_H_11_] (**8**)	6	2.76	2.22	1.73	21.9, 3.4, −1.8

**Table 2:** Skeletal electron pair (sep) count, selected structural parameters and ^11^B chemical shifts of **9**–**19**.

Metallaborane	sep	*d* [M-M] (Å)	*d*_avg_ [M-B] (Å)	*d*_avg_ [B-B] (Å)	^11^B NMR (ppm)
[(Cp*Cr)_2_B_4_H_8_Fe(CO)_3_] (**9**)	6	2.71	2.16	1.71	129.5, 105.8, 46.0, 4.1
[(Cp*Cr)_2_B_4_H_7_Co(CO)_3_] (**10**)	6	2.69	2.14	1.69	121.7, 113.9, 57.5, 36.6
[(Cp*Mo)_3_B_4_H_7_(µ-CO)_2_] (**11**)	6	2.83	2.23	1.72	88.8, 83.2, 46.3, 23.5
[(Cp*Mo)_2_B_4_H_6_W(CO)_5_] (**12**)	6	2.83	2.23	1.73	103.0 (2B), 77.9, 77.0
[(Cp*Mo)_2_B_4_H_8_W(CO)_4_] (**13**)	6	2.83	2.25	1.73	81.2 (2B), 41.9, 27.9
[(Cp*Mo)_2_B_4_H_8_Cr(CO)_4_] (**14**)	6	2.82	2.24	1.72	83.5, 81.3, 41.3, 27.1
[(Cp*W)_2_B_4_H_8_Cr(CO)_4_] (**15**)	6	2.82	2.26	1.75	70.6, 62.2, 36.5, 25.0
[(Cp*W)_2_B_4_H_8_Mo(CO)_4_] (**16**)	6	2.83	2.26	1.73	70.1, 60.1, 39.1, 25.6
[(Cp*W)_2_B_4_H_8_W(CO)_4_] (**17**)	6	2.83	2.25	1.71	70.3, 60.1, 35.5, 26.1
[(Cp*W)_2_B_4_H_8_Fe(CO)_3_] (**18**)	6	2.82	2.26	1.74	79.0, 76.2, 41.0, 35.8
[(Cp*W)_2_B_4_H_8_Ru(CO)_3_] (**19**)	6	2.82	2.27	1.75	77.7 (2B), 40.2, 34.0

**Table 3:** Skeletal electron pair count (sep), selected structural parameters, and ^11^B chemical shifts of **20**–**22**.

Metallaborane	sep	*d* [M-M] (Å)	*d_avg_* [M-B] (Å)	*d_avg_* [B-B] (Å)	^11^B NMR
[(Cp*Mo)_2_B_4_H_6_S] (**20**)	6	2.79	2.21	1.67	101.1, 76.7, 40.3, 10.1
[(Cp*Mo)_2_B_4_H_6_Se] (**21**)	6	2.78	2.24	1.83	100.7, 76.7, 41.8, 16.8
[(Cp*Mo)_2_B_4_H_5_PhSe] (**21-Ph**)	6	2.81	2.25	1.74	98.7, 77.5, 42.0, 28.2
[(Cp*Mo)_2_TeB_4_H_5_Cl] (**22-4Cl**)	6	2.84	2.17	1.69	95.5, 73.2, 40.7, 26.7
[(Cp*Mo)_2_TeB_4_H_5_Cl] (**22-3Cl**)	6	−	−	−	98.8, 71.8, 51.6, 24.7

**Table 4:** Skeletal electron pair count (sep), selected structural parameters and ^11^B NMR chemical shifts of **23**–**27**.

Metallaborane	sep	*d* [M-M] (Å)	*d_avg_* [M-B] (Å)	*d_avg_* [B-B] (Å)	^11^B NMR (ppm)
[(Cp*TaCl)_2_B_5_H_11_] (**23**)	7	3.22	2.38	1.77	77.7, 18.8, 15.0, −10.0
[(Cp*TaBr)_2_B_5_H_11_] (**24**)	7	3.23	2.38	1.76	81.1, 23.8, 22.4, −8.3
[(Cp*TaI)_2_B_5_H_11_] (**25**)	7	3.24	2.38	1.77	78.9, 25.8, 24.7, −11.9
[(Cp*TaCl)_2_B_5_H_10_Cl] (**26**)	7	3.23	2.37	1.76	79.2, 23.2, 19.8, 16.9, −10.0
[(Cp*W)_2_(TePh)B_5_H_5_(*µ*-H)_3_] (**27**)	7	2.85	2.32	1.74	64.9, 52.7, 9.4, −14.1

**Table 5:** Calculated bond length parameters (Å), Ta−Ta bond multiplicity, ^11^B chemical shifts (ppm), HOMO and LUMO energy (eV), and HOMO-LUMO gaps (ΔE, eV) for **23**–**25**.

Metallaborane	23	24	25
*d* [Ta-Ta]	3.25	3.25	3.26
*d_avg_* [B-B]	1.76	1.76	1.76
Ta−Ta bond multiplicity	0.23	0.23	0.23
^11^B NMR	10.0, 72.0, −14.4, 12.5, 12.5	14.1, 74.4, −14.2, 14.9, 14.5	20.8, 76.4, −15.2, 18.7, 18.0
HOMO	−4.629	−4.627	−4.555
LUMO	−2.629	−2.697	−2.832
ΔE	2.000	1.930	1.723

**Table 6:** Skeletal electron pair count (sep), selected structural parameters, and ^11^B chemical shifts of **28** and **29**.

Metallaborane	sep	*d_avg_* [M-B] (Å)	*d_avg_* [B-B] (Å)	^11^B NMR (ppm)
[(Cp_2_Zr)_2_B_5_H_11_] (**28**)	9	2.53	1.79	8.1, 2.5, −4.1
[(Cp_2_Hf)_2_B_5_H_11_] (**29**)	9	2.50	1.78	4.6, 2.0, −3.8
